# Antimicrobial Resistance in Urinary Tract Infections Among Patients with and Without Renal Comorbidities: A Retrospective Study from Al-Baha, Saudi Arabia

**DOI:** 10.3390/pathogens14121297

**Published:** 2025-12-17

**Authors:** Shazia Shaheen Mir, Eman Ali, Samiyah Ahmad Abdullah Alghamdi, Nora Mohamed Alghamdi, Raed A. Alharbi, Abdulmajeed A. A. Sindi, Ali A. Zaeri

**Affiliations:** Laboratory Medicine Department, Faculty of Applied Medical Sciences, Al-Baha University, Al-Baha 65779, Saudi Arabia

**Keywords:** urinary tract infection, antimicrobial resistance, uropathogens, comorbidities, *E. coli*, *K. pneumoniae*, Saudi Arabia

## Abstract

Urinary tract infections are among the most common bacterial infections worldwide, with increasing antimicrobial resistance posing a significant public health challenge. This study aimed to determine the demographic distribution, antimicrobial susceptibility patterns of uropathogens, and the clinical implications of UTIs in patients with renal comorbidities in the Al-Baha region of Saudi Arabia. A retrospective, cross-sectional study was conducted at King Fahad Hospital, Al-Baha, from January 2021 to September 2022. A total of 1126 culture-positive UTI cases were included. Patient demographics, uropathogen distribution, antimicrobial resistance profiles, and clinical characteristics were extracted from hospital records. Subgroup analysis was performed for 32 patients with renal comorbidities, including end-stage renal disease (ESRD), glomerulonephritis (GN), and kidney transplant recipients (KTs). Statistical analysis was performed using SPSS version 25. Most cases occurred in patients aged >70 years (43.2%) and females (68.29%). *Escherichia coli* (38.09%) and *Klebsiella pneumoniae* (14.02%) were the leading pathogens. High resistance to ampicillin (47–67%), cotrimoxazole (35–37%), and third-generation cephalosporins (34–47%) was observed, whereas carbapenems and aminoglycosides remained largely effective. Among the 32 patients with renal comorbidities, *E. coli* (43.8%), *Staphylococcus aureus* (25%), and *Enterococcus* spp. (18.8%) were the most common isolates. Dysuria (46.87%) and fever (31.25%) were the most frequent clinical presentations. Treatment regimens in this subgroup often required multidrug combinations, reflecting higher resistance burdens. Uropathogens in the Al-Baha region shows rising resistance to first-line antibiotics, with vulnerable populations such as patients with renal comorbidities experiencing distinct pathogen distributions and treatment challenges. Continuous surveillance, prudent antibiotic use, and targeted strategies for high-risk patients are essential to mitigate the impact of multidrug-resistant UTIs in Saudi Arabia.

## 1. Introduction

Urinary tract infections (UTIs) are among the most common bacterial infections globally, affecting individuals across age, gender, and health status [1]. Affecting both community and hospitalized populations, UTIs are responsible for significant morbidity and economic burden worldwide. Accounting for millions of healthcare visits and antibiotic prescriptions each year, UTIs present a significant burden on healthcare systems, particularly in developing regions and in populations with limited access to diagnostic tools and targeted treatment protocols [2,3]. The spectrum of UTIs ranges from uncomplicated infections such as cystitis to complicated cases, especially in patients with structural abnormalities or comorbid conditions like renal disease [4]. While UTIs are generally responsive to empirical antimicrobial therapy, the rising prevalence of antimicrobial resistance (AMR) among uropathogens has become a critical public health concern, limiting treatment options and increasing morbidity, mortality, and healthcare costs [5]. The global prevalence of UTIs varies based on geography, population demographics, and healthcare practices [6]. *Escherichia coli* remains the most frequently isolated pathogen, accounting for approximately 70–90% of community-acquired UTIs and a significant proportion of nosocomial infections [7]. In many low- and middle-resource regions, delayed diagnosis, empirical treatments, and inconsistent surveillance further complicate effective UTI management. In Saudi Arabia, the burden of UTIs is considerable, with multiple studies highlighting both the prevalence and the increasing complexity of cases due to AMR [1,7]. For instance, a cross-sectional study conducted in the Southwestern region by Alhazmi et al. (2023) reported that multidrug-resistant (MDR) strains were increasingly detected in both inpatient and outpatient settings, particularly among the elderly and those with comorbidities [8]. Another study carried out by Abalkhail et al. (2022) documented high rates of extended-spectrum β-lactamase (ESBL)-producing *E. coli*, emphasizing the growing threat of resistance to third-generation cephalosporins and fluoroquinolones [1]. These findings are echoed by Alanazi et al. (2022) and Alghamdi et al. (2023), who demonstrated a high prevalence of recurrent UTIs caused by ESBL-positive *E. coli* in a tertiary hospital, particularly among immunocompromised and elderly patients [6,9].

Surveillance data consistently show alarmingly high resistance rates to commonly used antibiotics such as ampicillin, cotrimoxazole, and ciprofloxacin. In a retrospective study conducted in Taif, Alhomayani et al. (2022) found that non-fermenting Gram-negative bacilli were emerging as prominent uropathogens, particularly in elderly and hospitalized patients with comorbidities [10]. Another multicenter study from the Madinah region by Almutawif and Eid (2023) confirmed rising resistance patterns to first-line antimicrobial agents, with carbapenems and aminoglycosides showing relative effectiveness against MDR strains [11]. Additionally, inappropriate antibiotic prescribing practices in emergency departments and primary care clinics have exacerbated the problem. Khalawi et al. (2024) analyzed antibiotic stewardship in a tertiary ED and found that up to 42% of prescriptions for UTIs were either unnecessary or deviated from guideline-recommended therapy [12]. Patients with chronic kidney disease (CKD), glomerulonephritis (GN), end-stage renal disease (ESRD), and kidney transplants (KT) are particularly vulnerable to UTIs. Structural and functional abnormalities of the urinary tract, immunosuppressive therapies, and frequent catheterization increase the risk of infection, recurrence, and complications. Bazaid et al. (2022) emphasized that comorbidities such as diabetes, CKD, and cardiovascular disease substantially elevate the risk of UTIs and MDR infections [13]. A meta-analysis by Ahmad et al. (2022) confirmed that the prevalence of UTIs among CKD patients is significantly higher than in the general population, with distinct uropathogen profiles and elevated resistance patterns [14]. Similarly, Mamari et al. (2022) found that immunocompromised patients, particularly those undergoing renal replacement therapy or immunosuppressive treatment post-transplant, had poorer clinical outcomes and required more aggressive multidrug regimens [15]. While national surveillance programs capture broad resistance patterns, granular data that integrate demographic, microbiological, and comorbidity-specific insights, especially from underserved regions like Al-Baha, are lacking.

Situated in the mountainous southwest, Al-Baha is one of the smaller administrative regions in Saudi Arabia and has a distinctive population demography with a very high proportion of elderly individuals and limited tertiary care infrastructure. Localized studies are crucial in such regions, where treatment strategies based on national data may not reflect on-the-ground realities. This knowledge gap is particularly concerning in the context of renal disease management, where recurrent infections and inappropriate antibiotic use can accelerate renal function decline. To address these gaps, the current study provides a focused retrospective analysis of 1126 culture-positive UTI cases from King Fahad Hospital in Al-Baha, Saudi Arabia, spanning from January 2021 to September 2022. The study aims to: (a) examine the demographic trends of UTI in the region, (b) identify the prevalence and antimicrobial resistance patterns of major uropathogens, and (c) explore the clinical and microbiological characteristics of patients with renal comorbidities such as end-stage renal disease (ESRD), glomerulonephritis (GN), and kidney transplants (KT). By providing robust, region-specific evidence, this research study contributes to the understanding of the UTI epidemiology in high-risk Saudi populations and underscores the need for targeted antimicrobial stewardship, surveillance, and tailored treatment protocols in secondary care settings.

## 2. Materials and Methods

### 2.1. Study Design and Setting

This retrospective, cross-sectional study was conducted at King Fahad Hospital (KFH), a tertiary-care referral center in the Al-Baha region of Saudi Arabia. The hospital provides specialized care to a wide catchment area, receiving referrals from regional primary health centres and private clinics. The study period spanned from January 2021 to September 2022, encompassing the late phase of the COVID-19 pandemic, when shifts in infection epidemiology and antimicrobial resistance (AMR) were anticipated.

### 2.2. Study Population and Sample Selection

The study included all adult patients (≥18 years) presenting with urinary tract infections (UTIs) confirmed by positive urine cultures during the study period. Both inpatients and outpatients of both genders were considered. Inclusion criteria were: (i) a culture-positive urine specimen identifying a uropathogenic bacterial or fungal isolate, (ii) complete antimicrobial susceptibility testing (AST) results, and (iii) cases recorded between January 2021 and September 2022. Exclusion criteria included: patients < 18 years, samples with mixed growths or negative cultures, and incomplete microbiological or clinical data.

From the total of 1126 culture-positive UTI cases, a subset of 32 patients was identified as having significant renal comorbidities, including end-stage renal disease (ESRD), glomerulonephritis (GN), and kidney transplant recipients (KT). This subgroup was analyzed separately to evaluate pathogen distribution, clinical characteristics, and therapeutic regimens compared to the general UTI population.

### 2.3. Data Collection

Data were extracted from the hospital’s OASIS electronic medical record (EMR) system using a standardized data collection form. Variables collected included: demographics (age, gender, nationality), clinical characteristics (inpatient/outpatient status, recurrence), comorbidity status (ESRD, GN, KT), and clinical presentation (fever, dysuria, urgency, frequency), microbiological findings (pathogen identification and distribution across groups), antimicrobial susceptibility (results of AST for each isolate), and therapeutic regimens (antibiotics prescribed for comorbid patients, including single- or multi-drug combinations).

### 2.4. Laboratory Methods

Urine samples were collected and processed in accordance with the hospital’s Internal Policy Procedures (IPP). Two different sample collection methods were used depending upon the patient’s clinical condition: midstream clean-catch urine and catheterized samples. All specimens were collected in sterile wide-mouth containers and submitted for culture based on the treating physician’s request. The urine cultures were performed under a safety cabinet using a 0.001 mL calibrated loop. Samples were inoculated onto 5% sheep blood agar, cysteine lactose electrolyte-deficient (CLED) agar, and MacConkey agar. Plates were incubated at 37 °C for 24–48 h in an incubator with 5.0% CO_2_ (Thermo Fisher Scientific, Waltham, MA, USA). A growth of ≥10^5^ colony-forming units (CFU)/mL was considered significant for infection, provided the patient also exhibited symptoms suggestive of UTI (such as dysuria, urgency, fever, or flank pain). In asymptomatic individuals or those with low colony counts, the clinical context and presence of pyuria or polymorphonuclear leukocytes were evaluated to differentiate asymptomatic bacteriuria from true infection.

Bacterial identification and antimicrobial susceptibility testing (AST) were conducted using the BD Phoenix™ M100 automated system (Becton, Dickinson and Company, Franklin Lakes, NJ, USA) [6]. A Gram stain was first performed to determine whether the isolate was Gram-positive or Gram-negative, guiding panel selection for AST. Colonies from overnight growth were suspended in ID broth and combined with AST indicators. After preparation, panels were scanned, loaded into the Phoenix instrument, and processed automatically. AST results were interpreted according to the Clinical and Laboratory Standards Institute (CLSI) 2021–2022 guidelines. Extended-spectrum β-lactamase (ESBL) production and carbapenem resistance in Gram-negative bacteria were confirmed using phenotypic confirmatory tests as per CLSI standards.

### 2.5. Statistical Analysis

Data were analyzed using IBM SPSS Statistics for Windows, Version 25.0 (IBM Corp., Armonk, NY, USA). Descriptive statistics (frequencies, percentages, means, and standard deviations) were used to summarize demographic and clinical data. Chi-square or Fisher’s exact tests were applied to examine associations between categorical variables such as patient status, comorbidities, pathogen distribution, and antibiotic resistance [6]. For the comorbidity subgroup (n = 32), pathogen profiles, clinical presentations, and treatment regimens were analyzed separately. A *p*-value of <0.05 was considered statistically significant.

## 3. Results

### 3.1. Demographic and Epidemiological Analysis

A total of 1126 urine culture-positive patients were included in this study. Of these, 769 (68.29%) were females, and 357 (31.71%) were males, indicating a higher prevalence of UTIs among females in this cohort. Most patients were Saudi nationals (93.90%), while non-Saudis accounted for 6.10%. Regarding the type of care, 637 patients (56.60%) were managed as outpatients, and 489 (43.40%) were admitted as inpatients. Nearly all cases were new episodes of UTI (99.90%), with only one case (0.09%) identified as a recurrence. Age-wise distribution revealed the predominance of elderly patients. The >70 years age group accounted for 486 cases (43.20%), making it the most affected group. The remaining age groups were more evenly distributed, ranging from 8.50% to 12.90%, with the 31–40 age group slightly higher at 12.90% (Figure S1; Supplementary Data). Chi-square analysis revealed a statistically significant association between gender and age group χ^2^(5) = 63.931, *p* < 0.001. Females represented a larger proportion of younger age groups (e.g., 84.10% of cases in the 31–40 group). There was also a significant association between gender and patient type χ^2^(1) = 11.252, *p* < 0.001. The proportion of males was higher among inpatients (37.01%) than among outpatients (27.63%). On the other hand, no significant associations were found between gender and nationality χ^2^(1) = 0.321, *p* = 0.571, or between gender and recurrence status χ^2^(1) = 0.465, *p* = 0.495 (Table 1).

**Figure 1:**
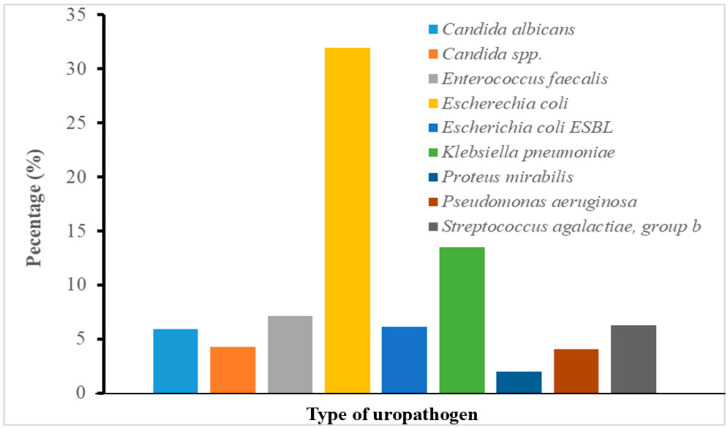
Distribution of major uropathogens isolated from urine cultures.

Most UTI cases in this study were diagnosed in the Emergency Clinic, accounting for 338 patients (30.02%), making it the most frequent point of care for initial UTI diagnosis. This was followed by the 2A Special Care Unit—Adult, with 159 patients (14.12%), and the Urology Clinic, with 50 patients (4.40%). The ICU (3A2—Intensive Care Unit) recorded 49 cases (4.30%), reflecting the burden of healthcare-associated infections in critical care settings. Other departments contributing significantly included the 5B Female Medical Ward (Pedia—Adult) with 62 cases (5.50%), Pediatrics (4A/4B) with a combined total of 69 cases (6.10%), and the Gynecology (7B) and OB-Gyne Clinic, contributing 28 (2.50%) and 31 (2.80%) cases, respectively (Table S1; Supplementary Data). UTI diagnoses were also observed in several outpatient specialty clinics, such as Urology Screening (31 cases, 2.80%), Nephrology (17 cases, 1.50%), and Haemodialysis Unit (10 cases, 0.90%). These figures reflect the wide departmental spectrum of UTI presentations, ranging from outpatient consultations to intensive inpatient care units. Among the different bacterial isolates, a total of 429 isolates (38.10%) were identified as *Escherichia coli*, making it the most frequently isolated uropathogen in this study. This includes both standard and variably labelled entries, such as *Escherichia coli* and *Escherichia coli* ESBL-positive (Figure 1). *Klebsiella pneumoniae* and its ESBL-positive variant represented 158 cases (14.00%), while *Enterococcus faecalis* accounted for 80 cases (7.10%). These were the second and third most common bacterial pathogens, respectively. Fungal organisms also constituted a significant proportion of isolates. *Candida albicans* species were identified in 67 cases (6.00%), while other *Candida* species were found in 48 cases (4.30%), indicating a combined fungal burden of 10.30%. Among Gram-positive organisms, *Streptococcus agalactiae* (group B) was found in 71 cases (6.30%), while coagulase-negative *Staphylococcus* species, including *S. epidermidis*, *S. haemolyticus*, *S. hominis*, and others, accounted for 56 isolates (5.00%). *Pseudomonas aeruginosa* was the predominant non-fermenting Gram-negative bacillus, isolated in 46 patients (4.10%), while *Proteus mirabilis* and *Enterobacter cloacae* were less common, representing 1.90% and 1.40%, respectively. The wide variety of bacterial and fungal isolates highlights the polymicrobial nature of UTIs with implications for empirical therapy and infection control.

### 3.2. Antimicrobial Resistance Patterns of Different Uropathogens

#### 3.2.1. Gram-Negative Bacteria

##### *Escherichia coli* 

*Escherichia coli* was the predominant uropathogen, and resistance patterns varied considerably across antibiotics (Table 2). In 2022, resistance was highest against ceftazidime (50.30%), cefepime (86.70%), ceftriaxone (46.60%), and ampicillin (47.10%), reflecting significant resistance to third- and fourth-generation cephalosporins and beta-lactams. Resistance to ciprofloxacin (37.40%) and cotrimoxazole (37.40%) was also notable, consistent with global trends. On the other hand, excellent sensitivity was preserved for amikacin (99.00%), imipenem (99.20%), meropenem (100.00%), and nitrofurantoin (92.40%), making these reliable treatment options. Moderate resistance was observed against gentamicin (10.40%) and tazobactam-piperacillin (13.70%). In 2021, broadly similar resistance patterns were observed, though with some key differences. Resistance to ampicillin (66.70%) and levofloxacin (44.50%) was higher in 2021 compared to 2022, while resistance to ceftazidime (27.40%) and cefepime (71.50%) was comparatively lower. Carbapenem resistance was slightly higher in 2021, with imipenem resistance (4.80%) and meropenem resistance (4.70%), compared to near-universal sensitivity in 2022. Overall, *E. coli* isolates demonstrated persistent high resistance to beta-lactams and cephalosporins, along with significant resistance to fluoroquinolones and cotrimoxazole, while maintaining excellent sensitivity to carbapenems, amikacin, and nitrofurantoin. The reduction in carbapenem resistance between 2021 and 2022 is noteworthy, whereas resistance to cephalosporins and fluoroquinolones remains a major clinical concern.

##### *Escherichia coli* ESBL

Among *E. coli* isolates, ESBL-producing strains exhibited very high resistance rates across most beta-lactams and cephalosporins, as expected (Table 3). In 2022, all isolates were resistant to ceftriaxone (100.00%), ceftazidime (100.00%), cefepime (100.00%), cefotaxime (100.00%), and cefuroxime (100.00%), confirming the ESBL phenotype. Resistance was also high to ciprofloxacin (83.30%), aztreonam (87.50%), ampicillin (100.00%), and norfloxacin (75.00%). On the other hand, strong activity was retained for carbapenems, with imipenem (96.90% sensitive) and meropenem (95.00% sensitive), as well as for amikacin (100.00% sensitive) and colistin (100.00% sensitive). Nitrofurantoin also showed good activity (75.00% sensitivity), and tazobactam-piperacillin retained moderate activity (85.70% sensitivity). In 2021, resistance patterns were similar. All isolates were resistant to ceftriaxone, cefepime, cefuroxime, and ceftazidime (100.00%), and resistance was high against ciprofloxacin (73.70%) and aztreonam (100.00%). Carbapenems remained effective, although sensitivity was slightly lower compared to 2022 (imipenem 89.50%, meropenem 87.50%). Aminoglycosides also retained reasonable activity, with amikacin (100.00% sensitive) and gentamicin (57.10% sensitive). Nitrofurantoin demonstrated an activity of 76.70%, and colistin remained fully active (100.00%). *E. coli* ESBL isolates demonstrated uniform resistance to cephalosporins and most beta-lactams, with high resistance to fluoroquinolones. Carbapenems, colistin, amikacin, and nitrofurantoin remained the most reliable treatment options, though the slight decline in carbapenem sensitivity in 2021 is clinically concerning.

##### *Klebsiella pneumoniae* 

*K. pneumoniae* isolates displayed marked resistance trends across multiple antibiotic classes with significant differences between 2021 and 2022 (Table 4). In 2022, resistance was particularly high to third- and fourth-generation cephalosporins: ceftriaxone (72.90%), cefotaxime (62.90%), ceftazidime (81.40%), cefuroxime (69.90%), and cefepime (94.40%). Resistance was also substantial for fluoroquinolones, with ciprofloxacin (59.70%) and levofloxacin (49.00%) showing reduced effectiveness. Carbapenem resistance was concerning, with imipenem (58.20%) and meropenem (63.60%) resistant isolates, limiting treatment options. By contrast, colistin (100.00% sensitive), tigecycline (100.00% sensitive), and gentamicin (94.80% sensitive) retained excellent activity, while amikacin (86.10% sensitive) and augmentin (65.80% sensitive) showed moderate efficacy. In 2021, the pattern was strikingly different. All isolates were reported as 100.00% sensitive to nearly all antibiotics tested, including cephalosporins, fluoroquinolones, beta-lactams, carbapenems, tigecycline, colistin, and aminoglycosides. Only ampicillin showed complete resistance (100.0%), which is consistent with the intrinsic resistance of *K. pneumoniae* to this agent. Overall, while *K. pneumoniae* isolates in 2021 were largely pan-sensitive (except for ampicillin), the 2022 isolates exhibited widespread multidrug resistance, particularly against cephalosporins, carbapenems, and fluoroquinolones. The preservation of activity for colistin, tigecycline, and aminoglycosides underscores their importance as last-resort therapies in managing multidrug-resistant *K. pneumoniae* infections.

##### *Klebsiella pneumoniae* ESBL

The ESBL-producing *K. pneumoniae* isolates exhibited widespread resistance to multiple antibiotic classes across both years, with limited therapeutic options available (Table 5). In 2022, all isolates were resistant to ceftriaxone (100.00%), cefotaxime (100.00%), and ceftazidime (100.00%), confirming the ESBL phenotype. Resistance was also high against cotrimoxazole (83.30%), ciprofloxacin (62.50%), and aztreonam (66.70%), while moderate resistance was noted for augmentin (60.00%). By contrast, strong activity was preserved for carbapenems, with imipenem (100.00% sensitive) and meropenem (100.00% sensitive). Amikacin (83.30% sensitive), gentamicin (75.00% sensitive), tazocin (100.00% sensitive), and nitrofurantoin (66.70% sensitive) also retained efficacy. In 2021, resistance patterns were similar. All isolates were resistant to ceftriaxone, cefotaxime, cefuroxime, and cefepime (100.00%), and fluoroquinolone resistance was high with ciprofloxacin (100.00%) and levofloxacin (100.00%). Nevertheless, carbapenems remained effective, with imipenem (80.00% sensitive) and meropenem (100.00% sensitive).

**Table 2:** Sensitivity patterns of *E. coli* for the years 2022 and 2021.

Tested Antibiotics	2022				2021			
	R	95% CI Resistant	S	95% CI Sensitive	R	95% CI Resistant	S	95% CI Sensitive
	N (%)		N (%)		N (%)		N (%)	
Cotrimoxazole	123 (37.38)	32.33–42.73	206 (62.62)	57.26–67.66	140 (35.26)	30.72–40.08	257 (64.74)	62.20–66.98
Ampicillin	105 (47.08)	40.63–53.63	118 (52.92)	49.55–56.17	62 (66.67)	56.59–75.41	31 (33.33)	29.19–38.79
Aztreonam	86 (42.57)	35.95–49.46	116 (57.43)	53.84–60.73	51 (37.78)	30.04–46.18	84 (62.22)	57.76–66.00
Meropenem	0 (0.00)	0	79 (100.00)	96.49–98.86	2 (4.65)	1.28–15.45	41 (95.35)	88.01–95.24
Norfloxacin	18 (31.58)	21.00–44.48	39 (68.42)	61.26–73.24	22 (22.00)	15.00–31.07	78 (78.00)	72.86–81.06
Imipenem	1 (0.76)	0.13–4.22	129 (99.24)	96.77–98.86	6 (4.76)	2.20–10.00	120 (95.24)	91.90–95.89
Amikacin	3 (1.00)	0.34–2.91	295 (99.00)	97.71–99.02	3 (0.89)	0.30–2.58	334 (99.11)	97.97–99.14
Ceftriaxone	123 (46.59)	40.66–52.61	141 (53.41)	50.31–56.40	110 (34.05)	29.10–39.38	213 (65.95)	63.13–68.38
Cefotaxime	40 (21.50)	16.20–27.95	146 (78.50)	74.92–80.91	32 (22.53)	16.43–30.07	110 (77.47)	73.26–80.22
Cefoxitin	0 (0.00)	0	8 (100.00)	75.50–92.05	-	-	-	-
Ceftazidime	98 (50.25)	43.30–57.20	97 (49.75)	46.20–53.29	29 (27.36)	19.77–36.52	77 (72.64)	67.57–76.12
Augmentin	27 (19.42)	13.70–26.78	112 (80.58)	76.41–83.08	37 (17.13)	12.69–22.71	179 (82.87)	79.738–84.85
Gentamicin	17 (10.43)	6.61–16.06	146 (89.57)	86.24–91.07	15 (4.81)	2.93–7.77	297 (95.19)	93.40–95.87
Nitrofurantoin	25 (7.60)	5.19–10.97	304 (92.40)	90.43–93.38	22 (5.35)	3.56–7.97	389 (94.65)	93.10–95.35
Cefuroxime	0 (0.00)	0	166 (100.00)	98.29–99.44	142 (34.55)	30.11–39.27	269 (65.45)	62.97–67.64
Ciprofloxacin	126 (37.38)	32.39–42.66	211 (62.62)	59.84–65.09	142 (33.33)	29.02–37.94	284 (66.667)	64.24–68.79
Cefepime	104 (86.66)	79.43–91.62	16 (13.34)	11.36–17.58	108 (71.52)	63.86–78.11	43 (28.48)	25.37–32.64
Colistin	0 (0.00)	0	3 (100.00)	57.60–86.24	7 (70.00)	39.67–8.22	3 (30.00)	22.91–48.18
Tazocin	31 (13.71)	9.83–18.81	195 (86.29)	83.38–87.96	7 (2.18)	1.06–4.43	314 (97.82)	96.39–98.11
Tigecycline	0 (0.00)	0	1 (100.00)	40.08–80.56	-	-	-	-
Levofloxacin	57 (21.75)	17.18–27.14	205 (78.25)	75.29–80.37	97 (44.49)	38.05–51.13	121 (55.51)	52.07–58.74

R = Resistance; S = Sensitivity; N = Number; % = Percentage; and CI = Class interval.

**Table 3:** Sensitivity patterns of ESBL *E. coli* for the years 2022 and 2021.

Tested Antibiotics	2022				2021			
	R	95% CI Resistant	S	95% CI Sensitive	R	95% CI Resistant	S	95% CI Sensitive
	N (%)		N (%)		N (%)		N (%)	
Cotrimoxazole	17 (58.62)	40.73–74.48	12 (41.38)	33.77–50.99	19 (55.88)	39.45–71.11	15 (44.12)	36.63–52.79
Ampicillin	5 (100.00)	56.55–100.00	0 (0.00)	0	3 (100.00)	43.84–100.00	0 (0.00)	0
Aztreonam	14 (87.50)	63.97–96.50	2 (12.50)	11.46–28.05	6 (100.00)	60.96–100.00	0 (0.00)	0
Meropenem	1 (5.00)	0.88–23.61	19 (95.00)	81.95–93.54	2 (12.50)	3.49–36.02	14 (87.50)	71.94–88.53
Norfloxacin	6 (75.00)	40.92–92.85	2 (25.00)	19.86–46.35	1 (33.33)	6.14–79.23	2 (66.67)	38.66–75.95
Imipenem	1 (3.12)	0.55–15.74	31 (96.87)	87.97–95.72	2 (10.53)	2.93–31.39	17 (89.47)	75.57–90.09
Amikacin	0 (0.00)	0	30 (100.00)	91.42–97.21	0 (0.00)	0	25 (100.00)	89.94–96.73
Ceftriaxone	19 (100.00)	83.18–100.00	0 (0.00)	0	9 (100.00)	70.08–100.00	0 (0.00)	7.32–22.58
Cefotaxime	2 (100.00)	34.23–100.00	0 (0.00)	0	-	-	-	-
Cefoxitin	2 (100.00)	34.23–100.00	0 (0.00)	0	-	-	-	-
Ceftazidime	16 (100.00)	80.63–100.00	0 (0.00)	0	1 (100.00)	20.65–100.00	0 (0.00)	0
Augmentin	20 (80.00)	60.86–91.13	5 (20.00)	16.27–31.71	5 (17.85)	7.87–35.59	23 (82.15)	71.19–85.33
Gentamicin	4 (33.33)	13.81–60.93	8 (66.67)	50.60–74.64	3 (42.86)	15.82–74.95	4 (57.14)	39.52–69.69
Nitrofurantoin	7 (25.00)	12.67–43.35	21 (75.00)	64.15–79.81	7 (23.33)	11.79–40.92	23 (76.67)	66.20–81.07
Cefuroxime	2 (100.00)	34.23–100.00	0 (0.00)	0	35 (100.00)	90.10–100.00	0 (0.00)	0
Ciprofloxacin	25 (83.33)	66.43–92.66	5 (16.67)	13.75–27.14	28 (73.68)	57.99–85.02	10 (26.32)	21.59–3.38
Cefepime	22 (100.00)	85.13–100.00	0 (0.00)	0	30 (100.00)	88.64–100.00	0 (0.00)	0
Colistin	0 (0.00)	0	1 (100.00)	40.08–80.56	0 (0.00)	0	5 (100.00)	67.19–89.35
Tazocin	2 (14.28)	4.00–33.94	12 (85.72)	68.85–87.19	1 (4.167)	0.73–20.24	23 (95.83)	84.53–94.48
Tigecycline	-	-	-	-	0 (0.00)	0	4 (100.00)	70.52–90.44
Levofloxacin	5 (100.00)	56.55–100.00	0 (0.00)	0	8 (66.67)	39.06–86.18	4 (33.33)	25.35–49.39

R = Resistance; S = Sensitivity; N = Number; % = Percentage; and CI = Class interval.

**Table 4:** Sensitivity patterns of *K. pneumoniae* for the years 2022 and 2021.

Tested Antibiotics	2022				2021			
	R	95% CI Resistant	S	95% CI Sensitive	R	95% CI Resistant	S	95% CI Sensitive
	N (%)		N (%)		N (%)		N (%)	
Cotrimoxazole	44 (47.31)	37.47–57.36	49 (52.69)	47.50–57.65	70 (50.00)	41.82–58.17	70 (50.00)	45.83–54.16
Ampicillin	1 (100.00)	20.65–100.00	0 (0.00)	0	26 (100.00)	87.12–100.00	0 (0.00)	0
Aztreonam	27 (56.25)	42.27–69.29	21 (43.75)	37.31–51.10	18 (58.06)	40.76–73.58	13 (41.94)	34.45–51.19
Meropenem	49 (63.63)	52.48–73.49	28 (36.37)	31.65–42.37	105 (78.95)	71.25–85.01	28 (21.05)	18.35–25.37
Norfloxacin	5 (55.55)	26.66–81.12	4 (44.45)	32.21–59.99	1 (7.69)	1.37–33.31	12 (92.31)	74.50–90.80
Imipenem	53 (58.24)	47.97–67.83	38 (41.76)	37.02–47.15	103 (69.13)	61.30–75.98	46 (30.87)	27.60–35.09
Amikacin	14 (13.86)	8.43–21.93	87 (86.14)	81.37–88.25	63 (36.42)	29.61–43.80	110 (63.58)	59.66–66.91
Ceftriaxone	78 (72.89)	63.79–80.41	29 (27.11)	23.65–32.13	0 (0.00)	0	45 (100.00)	94.06–98.07
Cefotaxime	22 (62.85)	46.33–76.83	13 (37.15)	30.63–46.19	0 (0.00)	0	23 (100.00)	89.19–96.49
Cefoxitin	1 (50.00)	9.45–90.54	1 (50.00)	29.31–70.68	-	-	-	-
Ceftazidime	57 (81.43)	70.77–88.81	13 (18.57)	15.60–24.80	0 (0.00)	0	23 (100.00)	89.19–94.49
Augmentin	13 (34.21)	21.21–50.10	25 (65.79)	56.96–71.71	0 (0.00)	0	53 (100.00)	94.89–98.34
Gentamicin	3 (5.17)	1.77–14.13	55 (94.83)	88.88–95.19	0 (0.00)	0	91 (100.00)	96.94–99.00
Nitrofurantoin	51 (60.71)	50.02–70.49	33 (39.29)	34.53–44.97	0 (0.00)	0	56 (100.00)	95.15–98.42
Cefuroxime	79 (69.91)	60.91–7.59	34 (30.09)	26.48–34.99	0 (0.00)	0	61 (100.00)	95.52–98.54
Ciprofloxacin	77 (59.68)	51.06–67.75	52 (40.32)	36.33–44.84	0 (0.00)	0	77 (100.00)	96.41–98.83
Cefepime	85 (94.44)	87.64–97.60	5 (5.56)	4.83–9.91	0 (0.00)	0	8 (100.00)	75.50–92.05
Colistin	0 (0.00)	0	57 (100.00)	95.23–98.45	0 (0.00)	0	111 (100.00)	97.47–99.18
Tazocin	45 (51.13)	40.86–61.31	43 (48.87)	43.69–54.12	0 (0.00)	0	90 (100.00)	96.90–98.99
Tigecycline	0 (0.00)	0	14 (100.00)	83.74–94.72	0 (0.00)	0	77 (100.00)	96.41–98.83
Levofloxacin	49 (49.00)	39.42–58.65	51 (51.00)	46.05–55.86	0 (0.00)	0	51 (100.00)	94.71–98.28

R = Resistance; S = Sensitivity; N = Number; % = Percentage; and CI = Class interval.

**Table 5:** Sensitivity patterns of ESBL *K. pneumoniae* for the years 2022 and 2021.

Tested Antibiotics	2022				2021			
	R	95% CI Resistant	S	95% CI Sensitive	R	95% CI Resistant	S	95% CI Sensitive
	N (%)		N (%)		N (%)		N (%)	
Cotrimoxazole	5 (83.33)	43.64–96.99	1 (16.67)	16.06–43.28	3 (100.00)	43.84–10000	0 (0.00)	0
Ampicillin	-	-	-	-	-	-	-	-
Aztreonam	2 (66.67)	20.76–93.85	1 (33.33)	24.04–61.33	1 (100.00)	20.65–100.00	0 (0.00)	0
Meropenem	0 (0.00)	0	2 (100.00)	50.34–83.89	0 (0.00)	0	2 (100.00)	50.34–83.8
Norfloxacin	-	-	-	-	-	-	-	-
Imipenem	0 (0.00)	0	7 (100.00)	73.24–91.32	1 (20.00)	3.62–62.44	4 (80.00)	51.95–81.97
Amikacin	1 (16.67)	3.00–56.35	5 (83.33)	56.71–83.93	1 (25.00)	4.55–69.93	3 (75.00)	46.07–79.43
Ceftriaxone	4 (100.00)	51.00–100.00	0 (0.00)	0	3 (100.00)	43.84–100.00	0 (0.00)	0
Cefotaxime	2 (100.00)	34.23–100.00	0 (0.00)	0	2 (100.00)	34.23–100.00	0 (0.00)	0
Cefoxitin	-	-	-	-	-	-	-	-
Ceftazidime	2 (100.00)	34.23–100.00	0 (0.00)	0	-	-	-	-
Augmentin	3 (60.00)	23.07–88.23	2 (40.00)	27.72–60.96	1 (33.33)	6.14–79.23	2 (66.67)	38.66–75.95
Gentamicin	1 (25.00)	4.55–69.93	3 (75.00)	46.07–79.43	0 (0.00)	0	2 (100.00)	50.34–83.89
Nitrofurantoin	1 (33.33)	6.14–79.23	2 (66.67)	38.66–75.95	1 (50.00)	9.45–90.54	1 (50.00)	29.31–70.68
Cefuroxime	-	-	-	-	3 (100.00)	43.84–100.00	0 (0.00)	0
Ciprofloxacin	5 (62.50)	30.57–86.31	3 (37.50)	27.33–55.75	5 (100.00)	56.55–100.00	0 (0.00)	0
Cefepime	3 (50.00)	18.76–81.23	3 (50.00)	34.06–65.93	3 (100.00)	43.84–100.00	0 (0.00)	0
Colistin	-	-	-	-	1 (100.00)	20.65–100.00	0 (0.00)	0
Tazocin	0 (0.00)	0	3 (100.00)	57.60–86.24	1 (100.00)	20.65–100.00	0 (0.00)	0
Tigecycline	-	-	-	-	0 (0.00)	0	1 (100.00)	40.08–80.56
Levofloxacin	-	-	-	-	1 (100.00)	20.65–100.00	0 (0.00)	19.43–59.91

R = Resistance; S = Sensitivity; N = Number; % = Percentage; and CI = Class interval.

Aminoglycosides retained partial activity, with amikacin (75.00% sensitive) and gentamicin (100.00% sensitive). Nitrofurantoin showed variable activity, with 50.00% sensitivity. Overall, *K. pneumoniae* ESBL isolates displayed universal resistance to cephalosporins and most beta-lactams, alongside high resistance to fluoroquinolones. Carbapenems, amikacin, tazocin, and colistin (where tested) remained the most reliable therapeutic options, though the presence of partial resistance even among carbapenems (2021) is concerning.

#### 3.2.2. Gram-Positive Bacteria

##### *Staphylococcus* Species

*Staphylococcus* species isolates were tested against multiple antibiotics for the years 2021 and 2022, and the results revealed marked variation in susceptibility patterns (Table 6). In 2022, high resistance was observed to imipenem (93.30%) and ampicillin (70.00%), whereas resistance to gentamicin (3.10%), cotrimoxazole (12.50%), and ciprofloxacin (12.50%) was comparatively low. Importantly, all isolates remained sensitive to linezolid (100.00%) and nitrofurantoin (100.00%). Moderate resistance was noted for oxacillin (56.30%) and augmentin (47.40%), reflecting ongoing methicillin resistance trends. Similar resistance patterns were observed in 2021, with high resistance to ampicillin (84.40%) and imipenem (85.50%), while lower resistance was seen for gentamicin (9.30%), ciprofloxacin (27.00%), and cotrimoxazole (2.40%). All isolates in 2021 were sensitive to linezolid (100.00%) and slightly less sensitive to nitrofurantoin (96.60%) and gentamicin (90.70%). The resistance to beta-lactams (ampicillin, oxacillin, cephalexin, and imipenem) remained consistently high, whereas sensitivity was preserved for linezolid, nitrofurantoin, gentamicin, and ciprofloxacin. A concerning finding was the sharp increase in resistance to vancomycin from 0.00% (2021) to 37.40% (2022), reflecting the emergence of vancomycin-resistant *staphylococci*.

##### Methicillin-Resistant *Staphylococcus aureus* (MRSA)

MRSA isolates were tested during 2021 and 2022. The resistance profile demonstrated that beta-lactam antibiotics (ampicillin, imipenem, oxacillin, augmentin, cefotaxime) showed 100.00% resistance in both years, consistent with the expected resistance phenotype of MRSA (Table 7). Encouragingly, all MRSA isolates remained fully sensitive to linezolid and nitrofurantoin in both 2021 and 2022. Similarly, susceptibility to vancomycin was maintained, with 100.00% sensitivity in 2022 and 88.90% in 2021, though the presence of two resistant isolates in 2021 (11.10%) is of clinical concern. Other antibiotics demonstrated variable activity. In 2022, all isolates were fully sensitive to cotrimoxazole (100.00%), ciprofloxacin (100.00%), and clindamycin (100.00%), while resistance to gentamicin was modest (33.30%). In 2021, moderate resistance was noted against ciprofloxacin (100.00%), gentamicin (40.00%), and clindamycin (16.70%), whereas cotrimoxazole retained full sensitivity. MRSA isolates exhibited predictable beta-lactam resistance, but continued to show excellent sensitivity to linezolid, nitrofurantoin, cotrimoxazole, and vancomycin.

##### *Streptococcus* Species

Antibiotic susceptibility testing of *Streptococcus* isolates from 2021 and 2022 revealed generally favourable sensitivity profiles with some variability across antibiotic classes (Table 8). In 2022, isolates demonstrated high sensitivity to beta-lactams, with 100.00% susceptibility reported for cephalexin, cefazolin, amoxicillin, cefotaxime, augmentin, nitrofurantoin, and penicillin, while resistance was negligible for ampicillin (2.20%). Full sensitivity was also maintained for vancomycin (100.00%). However, notable resistance was observed for gentamicin (33.30%) and ciprofloxacin (41.70%), suggesting reduced efficacy of aminoglycosides and fluoroquinolones against these isolates. In 2021, the susceptibility pattern was similar, with nearly universal sensitivity to beta-lactams (ampicillin, cephalexin, cefotaxime, augmentin, nitrofurantoin, penicillin, amoxicillin). Resistance remained minimal (≤2.00%) for most of these agents. However, higher resistance was recorded for gentamicin (58.30%), whereas ciprofloxacin resistance was only 5.60%, indicating a sharp increase in fluoroquinolone resistance between 2021 and 2022. All isolates were fully sensitive to clindamycin (100.00%) and vancomycin (100.00%). Overall, *Streptococcus* spp. isolates retained excellent sensitivity to beta-lactams and glycopeptides, but demonstrated emerging resistance to fluoroquinolones and aminoglycosides, highlighting the need for cautious use of these agents in empirical therapy.

#### 3.2.3. *Enterococcus* Species

The susceptibility profile of *Enterococcus* species showed variation across the two years, with concerning resistance to some key drugs (Table 9). In 2022, isolates demonstrated excellent susceptibility to vancomycin (100.00%), ampicillin (93.50%), amoxicillin (97.60%), imipenem (97.20%), and linezolid (100.00%). Amikacin also retained complete activity (100.0%). However, high resistance rates were observed for gentamicin (83.30%) and clindamycin (85.70%), indicating limited effectiveness of these agents. Moderate resistance was noted for ciprofloxacin (46.50%), penicillin (14.30%), and moxifloxacin (33.30%), while nitrofurantoin maintained high activity (93.30% sensitive). In 2021, susceptibility was somewhat lower. Vancomycin resistance was 15.80%, raising concern for the presence of VRE. Resistance to ampicillin (13.00%), amoxicillin (12.60%), and imipenem (16.70%) was higher compared to 2022.

**Table 6:** Sensitivity patterns of *Staphylococcus* species for the years 2022 and 2021.

Tested Antibiotics	2022				2021			
	R	95% CI Resistant	S	95% CI Sensitive	R	95% CI Resistant	S	95% CI Sensitive
	N (%)		N (%)		N (%)		N (%)	
Vancomycin	123 (37.38)	37.75–42.98	206 (62.61)	57.60–68.06	0 (0.00)	0	56 (100.00)	96.68–103.31
Cotrimoxazole	5 (12.50)	16.51–27.31	35 (87.50)	77.273–98.87	2 (2.41)	4.61–8.55	81 (97.59)	93.70–101.58
Cephalexin	1 (10.00)	24.50–46.93	9 (90.00)	69.18–114.03	36 (81.82)	82.58–94.27	8 (18.18)	9.91–33.30
Cefazolin	1 (33.33)	59.35–110.16	2 (66.67)	28.87–130.48	-	-	-	-
Ampicillin	14 (70.00)	72.63–92.94	6 (30.00)	15.82–56.44	54 (84.37)	84.83–93.94	10 (15.62)	8.97–27.20
Rifampicin	0 (0.00)	0	3 (100.00)	60.96–139.03	2 (9.09)	16.39–30.05	20 (90.91)	77.98–105.29
Amoxicillin	4 (44.44)	54.21–86.23	5 (55.55)	31.35–95.39	17 (62.96)	65.42–83.68	10 (37.03)	22.96–59.47
Imipenem	14 (93.33)	94.09–110.03	1 (6.66)	1.32–33.20	47 (85.45)	85.94–95.56	8 (14.54)	7.81–27.04
Cefotaxime	7 (38.89)	44.78–67.30	11 (61.11)	42.34–87.40	6 (23.07)	28.37–44.94	20 (76.92)	61.93–95.08
Oxacillin	18 (56.25)	58.72–75.90	14 (43.75)	29.76–64.11	55 (65.47)	66.25–76.43	29 (34.52)	25.79–46.17
Augmentin	18 (47.37)	49.90–65.76	20 (52.63)	39.05–70.77	58 (59.18)	59.96–69.70	40 (40.82)	32.21–51.69
Gentamicin	1 (3.12)	8.61–16.63	31 (96.87)	89.02–105.07	7 (9.33)	11.59–18.48	68 (90.67)	84.01–97.78
Penicillin	2 (100.00)	100.00–148.99	0 (0.00)	0	6 (100.00)	100.00–124.25	0 (0.00)	0.00
Nitrofurantoin	0 (0.00)	0	42 (100.00)	95.62–104.37	3 (3.37)	5.41–9.64	86 (96.63)	92.46–100.93
Ciprofloxacin	4 (12.50)	17.45–29.65	28 (87.50)	76.00–100.41	20 (27.02)	28.87–39.05	54 (72.97)	63.47–83.83
Clindamycin	3 (30.00)	41.28–70.04	7 (70.00)	46.07–103.60	7 (41.17)	47.15–70.49	10 (58.82)	39.66–86.37
Moxifloxacin	1 (100.00)	100.00–165.76	0 (0.00)	0	1 (2.13)	5.97–11.50	46 (97.87)	92.37–103.53
Colistin	-	-	-	-	-	-	-	-
Linezolid	0 (0.00)	0	17 (100.00)	89.84–110.15	0 (0.00)	0	58 (100.00)	96.79–103.20
Doxycycline	-	-	-	-	2 (100.00)	100.00–148.99	0 (0.00)	0.000

R = Resistance; S = Sensitivity; N = Number; % = Percentage; and CI = Class interval.

**Table 7:** Sensitivity patterns of MRSA species for the years 2022 and 2021.

Tested Antibiotics	2022				2021			
	R	95% CI Resistant	S	95% CI Sensitive	R	95% CI Resistant	S	95% CI Sensitive
	N (%)		N (%)		N (%)		N (%)	
Vancomycin	0 (0.00)	0	7 (100.00)	78.46–121.53	2 (11.11)	19.68–35.96	16 (88.89)	73.67–106.24
Cotrimoxazole	0 (0.00)	0	6 (100.00)	75.75–124.25	0 (0.00)	0	14 (100.00)	87.93–112.06
Cephalexin	-	-	-	-	5 (100.00)	100.00–127.75	0 (0.00)	0
Cefazolin	-	-	-	-	-	-	-	-
Ampicillin	7 (100.00)	100.00–121.53	0 (0.00)	0	9 (100.00)	100.00–117.58	0 (0.00)	0
Rifampicin	-	-	-	-	0 (0.00)	0	3 (100.00)	60.96–139.03
Amoxicillin	2 (100.00)	100.00–148.99	0 (0.00)	0	1 (100.00)	100.00–165.76	0 (0.00)	0
Imipenem	7 (100.00)	100.00–121.53	0 (0.00)	0	10 (100.00)	100.00–116.11	0 (0.00)	0
Cefotaxime	2 (100.00)	100.00–148.99	0 (0.00)	0	3 (100.00)	100.00–139.03	0 (0.00)	0
Oxacillin	7 (100.00)	100.00–121.53	0 (0.00)	0	13 (100.00)	100.00–112.87	0 (0.00)	0
Augmentin	8 (100.00)	100.00–119.36	0 (0.00)	0	15 (100.00)	100.00–111.35	0 (0.00)	0
Gentamicin	2 (33.33)	49.50–86.97	4 (66.67)	37.27–112.22	2 (40.00)	56.65–98.27	3 (60.00)	29.47–112.72
Penicillin	1 (100.00)	100.00–165.76	0 (0.00)	0	1 (100.00)	100.00–165.76	0 (0.00)	0
Nitrofurantoin	0 (0.00)	0	7 (100.00)	78.46–121.53	0 (0.00)	0	10 (100.00)	83.88–116.11
Ciprofloxacin	0 (0.00)	0	7 (100.00)	78.46–121.53	6 (100.00)	100.00–124.25	0 (0.00)	0
Clindamycin	0 (0.00)	0	1 (100.00)	34.23–165.76	1 (16.67)	36.87–70.01	5 (83.33)	54.23–120.51
Moxifloxacin	-	-	-	-	3 (37.50)	49.60–82.86	5 (62.50)	36.49–103.02
Colistin	-	-	-	-	-	-	-	-
Linezolid	0 (0.00)	0	7 (100.00)	78.46–121.53	0 (0.00)	0	7 (100.00)	78.46–121.53
Doxycycline	-	-	-	-	1 (100.00)	100.00–165.76	0 (0.00)	0

R = Resistance; S = Sensitivity; N = Number; % = Percentage; and CI = Class interval.

**Table 8:** Sensitivity patterns of *Staphylococcus* species for the years 2022 and 2021.

Tested Antibiotics	2022				2021			
	R	95% CI Resistant	S	95% CI Sensitive	R	95% CI Resistant	S	95% CI Sensitive
	N (%)		N (%)		N (%)		N (%)	
Vancomycin	0 (0.00)	0	1 (100.00)	34.23–165.76	0 (0.00)	0	1 (100.00)	34.23–165.76
Cotrimoxazole	4 (25.00)	33.04–58.80	12 (75.00)	55.91–99.44	5 (22.73)	28.93–46.92	17 (77.27)	61.10–97.09
Cephalexin	0 (0.00)	0	9 (100.00)	82.41–117.58	0 (0.00)	0	30 (100.00)	93.98–106.01
Cefazolin	0 (0.00)	0	3 (100.00)	60.96–139.03	-	-	-	-
Ampicillin	1 (2.18)	6.09–11.79	45 (97.82)	92.21–103.60	0 (0.00)	0	71 (100.00)	97.36–102.63
Rifampicin	-	-	-	-	-	-	-	-
Amoxicillin	0 (0.00)	0	43 (100.00)	95.72–104.27	0 (0.00)	0	59 (100.00)	96.84–103.15
Imipenem	0 (0.00)	0	1 (100.00)	34.23–165.76	-	-	-	-
Cefotaxime	0 (0.00)	0	31 (100.00)	94.16–105.83	0 (0.00)	0	27 (100.00)	93.35–106.64
Oxacillin	0 (0.00)	0	2 (100.00)	51.01–148.99	1 (50.00)	74.49–134.90	1 (50.00)	14.08–134.90
Augmentin	0 (0.00)	0	47 (100.00)	96.07–103.92	0 (0.00)	0	76 (100.00)	97.53–102.46
Gentamicin	5 (33.33)	40.90–64.90	10 (66.67)	46.44–94.45	7 (58.33)	64.08–91.80	5 (41.67)	21.99–77.43
Penicillin	0 (0.00)	0	44 (100.00)	95.81–104.18	1 (1.61)	4.56–8.84	61 (98.39)	94.16–102.71
Nitrofurantoin	0 (0.00)	0	32 (100.00)	94.33–105.66	1 (1.61)	4.56–8.84	61 (98.39)	94.16–102.71
Ciprofloxacin	5 (41.67)	49.71–77.43	7 (58.33)	36.35–91.80	1 (5.56)	14.66–28.24	17 (94.44)	81.40–108.55
Clindamycin	1 (14.28)	32.74–62.36	6 (85.72)	59.17–118.41	0 (0.00)	0	17 (100.00)	89.84–110.15
Moxifloxacin	-	-	-	-	0 (0.00)	0	2 (100.00)	51.01–148.99
Colistin	-	-	-	-	-	-	-	-
Linezolid	-	-	-	-	-	-	-	-
Doxycycline	-	-	-	-	-	-	-	-

R = Resistance; S = Sensitivity; N = Number; % = Percentage; and CI = Class interval.

**Table 9:** Sensitivity patterns of *Enterococcus* species for the years 2022 and 2021.

Tested Antibiotics	2022				2021			
	R	95% CI Resistant	S	95% CI Sensitive	R	95% CI Resistant	S	95% CI Sensitive
	N (%)		N (%)		N (%)		N (%)	
Vancomycin	0 (0.00)	0	7 (100.00)	78.46–121.53	9 (15.79)	18.53–28.25	48 (84.21)	75.00–94.44
Cotrimoxazole	4 (50.00)	59.68–93.67	4 (50.00)	25.68–93.67	30 (100.00)	100.00–106.01	0 (0.00)	0
Cephalexin	-	-	-	-	-	-	-	-
Cefazolin	-	-	-	-	-	-	-	-
Ampicillin	3 (6.52)	10.26–18.20	43 (93.48)	85.80–101.67	16 (13.00)	14.34–20.39	107 (87.00)	81.14–93.24
Rifampicin	-	-	-	-	1 (25.00)	49.33–92.62	3 (75.00)	39.81–126.40
Amoxicillin	1 (2.38)	6.65–12.86	41 (97.62)	91.51–103.93	11 (12.64)	14.53–21.69	76 (87.36)	80.46–94.79
Imipenem	1 (2.78)	7.702–14.88	35 (97.22)	90.17–104.54	12 (16.67)	18.83–27.61	60 (83.33)	74.98–92.54
Cefotaxime	-	-	-	-	2 (100.00)	100.00–148.99	0 (0.00)	0
Oxacillin	1 (100.00)	100.00–165.76	0 (0.00)	0	1 (100.00)	100.00–165.76	0 (0.00)	0
Augmentin	1 (2.27)	6.36–12.30	43 (97.73)	91.88–103.76	15 (13.16)	14.59–20.92	99 (86.84)	80.73–93.38
Gentamicin	5 (83.33)	87.37–120.51	1 (16.67)	3.73–70.01	14 (82.35)	84.14–103.33	3 (17.65)	6.82–45.19
Penicillin	4 (14.28)	19.78–33.51	24 (85.72)	72.90–100.35	4 (40.00)	49.66–79.80	6 (60.00)	36.30–96.58
Nitrofurantoin	3 (6.67)	10.48–18.58	42 (93.33)	85.50–101.70	12 (10.53)	12.00–17.79	102 (89.47)	83.86–95.43
Ciprofloxacin	20 (46.51)	48.79–63.69	23 (53.49)	40.57–70.37	45 (47.37)	48.41–58.45	50 (52.63)	43.53–63.61
Clindamycin	6 (85.71)	88.79–118.41	1 (14.29)	3.12–62.36	11 (91.67)	92.81–112.10	1 (8.33)	1.69–40.27
Moxifloxacin	1 (33.33)	59.35–110.16	2 (66.67)	28.87–130.48	21 (37.50)	39.57–53.27	35 (62.50)	51.04–76.44
Colistin	-	-	-	-	-	-	-	-
Linezolid	0 (0.00)	0	2 (100.00)	51.01–148.99	0 (0.00)	17.58–35.17	9 (100.00)	82.41–117.58
Doxycycline	50 (16.07)	16.59–20.69	261 (83.93)	79.91–88.12	1 (14.29)	32.74–62.36	6 (85.71)	59.17–118.41
Levofloxacin	-	-	-	-	-	-	-	-
Cotrimoxazole	7 (100.00)	100.00–121.53	0 (0.00)	0	-	-	-	-
Ampicillin	-	-	-	-	-	-	-	-
Aztreonam	1 (3.70)	10.09–19.49	26 (96.30)	87.14–105.94	2 (5.71)	10.61–19.57	33 (94.29)	85.62–103.53
Meropenem	2 (50.00)	66.22–112.57	2 (50.00)	19.87–112.57	5 (41.67)	49.71–77.43	7 (58.33)	36.35–91.80
Norfloxacin	-	-	-	-	0 (0.00)	0	2 (100.00)	51.01–148.99
Imipenem	1 (7.69)	19.57–37.60	12 (92.31)	75.27–111.32	7 (21.87)	26.29–40.95	25 (78.13)	64.71–94.01
Amikacin	0 (0.00)	0	34 (100.00)	94.65–105.34	5 (9.61)	12.83–21.34	47 (90.39)	82.21–99.23
Ceftriaxone	-	-	-	-	1 (100.00)	100.00–165.76	0 (0.00)	0
Cefotaxime	1 (100.00)	100.00–165.76	0 (0.00)	0	-	-	-	-
Cefoxitin	-	-	-	-	-	-	-	-
Ceftazidime	2 (6.06)	11.22–20.68	31 (93.94)	84.81–103.72	12 (23.53)	26.30–38.09	39 (76.47)	65.53–89.11
Augmentin	4 (100.00)	100.00–132.44	0 (0.00)	0	1 (100.00)	100.00–165.76	0 (0.00)	0
Gentamicin	0 (0.00)	0	14 (100.00)	87.93–112.06	4 (13.79)	19.14–32.45	25 (86.21)	73.75–100.37
Nitrofurantoin	3 (100.00)	100.00–139.03	0 (0.00)	0	-	-	-	-
Cefuroxime	-	-	-	-	-	-	-	-
Ciprofloxacin	3 (9.09)	14.09–24.87	30 (90.91)	80.63–102.18	12 (22.22)	24.89–36.13	42 (77.78)	67.29–89.78
Cefepime	2 (6.89)	12.68–23.32	27 (93.11)	82.88–104.18	7 (17.95)	21.80–34.20	32 (82.05)	70.49–95.29
Colistin	0 (0.00)	0	3 (100.00)	60.96–139.03	0 (0.00)	0	9 (100.00)	82.41–117.58
Tazocin	0 (0.00)	0	28 (100.00)	93.58–106.42	1 (2.38)	6.65–12.86	41 (97.62)	91.51–103.93
Tigecycline	-	-	-	-	-	-	-	-
Levofloxacin	3 (11.11)	17.01–29.92	24 (88.89)	76.72–102.53	6 (17.14)	21.45–34.38	29 (82.86)	70.82–96.67

R = Resistance; S = Sensitivity; N = Number; % = Percentage; and CI = Class interval.

Resistance to ciprofloxacin (47.40%) and moxifloxacin (37.50%) was comparable to 2022. Aminoglycoside resistance remained high, with gentamicin (82.40% resistant). Linezolid maintained 100.00% activity. *Enterococcus* isolates showed excellent sensitivity to vancomycin (100.00%), linezolid (100.00%), amoxicillin (97.62%), and imipenem (97.22%), especially in 2022, while maintaining high-level resistance to oxacillin (100.00%), cotrimoxazole (100.00%), nitrofurantoin (100.00%), cefotaxime (100.00%), gentamicin (83.33%) and clindamycin (85.71%). The detection of vancomycin resistance (15.80% sensitivity) in 2021 is of clinical concern, though no resistant isolates were observed in 2022.

### 3.3. UTI Patients with Renal Comorbidities

Out of the 1126 culture-positive urine samples analyzed, 32 patients (2.84%) were identified to have underlying renal comorbidities, including kidney transplantation (KT), glomerulonephritis (GN), and end-stage renal disease (ESRD). This subgroup was evaluated separately due to their increased vulnerability to multidrug-resistant infections and atypical clinical presentations. These patients were analyzed for their demographic distribution (age), pathogen distribution (bacteria and fungi), clinical presentation (fever, urgency, frequency, and dysuria), and antibiotic resistance trends/patterns.

#### 3.3.1. Age Distribution of UTI in Patients with Renal Comorbidities

Age distribution analysis showed that most of these cases were within the 41–50 years age group (9; 28.12%), followed by patients aged 18–30 years (8; 25.00%) and >70 years (7; 21.87%). Smaller proportions of cases were seen in the 31–40 years (2; 6.25%), 51–60 years (2; 6.25%), and 61–70 years (4; 12.50%) age groups (Table 10). Among the sub-categories, ESRD patients constituted the majority (56.26%), while KT and GN cases were comparatively fewer but equally represented the comorbidity (21.87%). Chi-square analysis revealed no statistically significant association between patient age groups and the type of renal comorbidity (χ^2^ = 16.90, df = 10, *p* = 0.076), although a near-significant trend was observed. ESRD was more common in older patients, whereas KT patients were more represented in younger age groups.

#### 3.3.2. Microbiological and Clinical Profile of UTI Patients with Renal Comorbidities

Among the culture-positive cases with renal comorbidities (32), Gram-negative organisms were isolated in 50.00% of patients, Gram-positive bacteria in 43.75%, and *Candida* spp. in 6.25% (Table 11). Among Gram-negative bacteria, *Escherichia coli* was the most prevalent pathogen (43.75% of isolates), affecting patients across all comorbidity groups (35.71% in KT, 21.43% in GN, and 42.86% in ESRD). Among Gram-positive organisms, *Staphylococcus aureus* was most frequently detected (25.00% of isolates), predominating in ESRD patients (75.00%). Additionally, *Staphylococcus aureus* was closely followed by *Enterococcus faecium* (18.75%). Less frequent isolates included *Klebsiella pneumoniae* and *Pseudomonas aeruginosa* (3.12%) and *Candida* spp. (6.25%). Among the comorbidities sub-groups, ESRD patients accounted for most isolates (56.26%), while KT and GN cases contributed equally (21.87%). No significant association was found between the type of comorbidity and the isolated uropathogen (χ^2^ = 10.13, df = 10, *p* = 0.429). However, descriptive trends indicated that *Escherichia coli* predominated across all comorbidities, while *Staphylococcus* and *Enterococcus* species were more frequent in ESRD cases.

The clinical manifestations of UTI in patients with renal comorbidities are summarized in Table 12. Analysis of clinical presentation across comorbidity groups (KT, GN, ESRD) revealed dysuria was the most frequently reported clinical manifestation, present in 46.87% of cases, followed by fever (31.25%), frequency (12.50%), and urgency (9.37%). However, no statistically significant associations (Fisher’s exact) were found between specific symptoms and underlying renal comorbidities (all *p* > 0.05). Although fever was more frequently observed in GN patients (40.00%), the association did not reach statistical significance (OR = 4.22, *p* = 0.165). Dysuria remained the most prevalent symptom overall (15 cases, 46.87% of cases), particularly among ESRD patients (60.00%), but again without significant association (*p* > 0.05). Urinary frequency (12.50%) and urgency (9.37%) were observed less frequently. Among the renal comorbidities sub-groups ESRD patients accounted for more than half of the symptomatic cases (56.26%), while KT and GN patients contributed 21.87% each. These findings suggest that while symptom distribution varies across comorbidity types, the differences were not strong enough to demonstrate statistical significance in this sample.

#### 3.3.3. Antibiotics Treatment Patterns in Comorbidity-Associated UTI

Among the 32 UTI-positive patients with renal comorbidities, *Escherichia coli* was the most frequently isolated pathogen, identified in 14 cases (43.75%). The distribution across comorbid conditions showed five cases in kidney transplant recipients (35.71%), three in patients with glomerulonephritis (21.42%), and six in end-stage renal disease (42.85%) (Table 13). Aminoglycoside-based antibiotic regimens formed the cornerstone of treatment across all subgroups. In kidney transplant recipients, the majority (80.00%) were treated with aminoglycoside–cephalosporin combinations or aminoglycoside alone, with only one patient receiving ampicillin monotherapy. In the glomerulonephritis group, treatment included aminoglycoside–carbapenem combinations and ampicillin. Patients with ESRD demonstrated the greatest therapeutic diversity, with regimens that incorporated aminoglycosides, cephalosporins, carbapenems, and fluoroquinolones. Collectively, aminoglycoside-centred therapies accounted for more than two-thirds of all treatment regimens for *E. coli*.

*S. aureus* accounted for eight cases (25.00%) of UTI among patients with renal comorbidities (Table 14). Of these, one case was identified in a kidney transplant recipient (12.50%), one in glomerulonephritis (12.50%), and six in ESRD (75.00%). The kidney transplant recipient was treated with an aminoglycoside–cephalosporin combination, while the GN patient received an aminoglycoside–carbapenem regimen. In contrast, ESRD patients exhibited a wide range of treatment regimens, including aminoglycoside combinations (such as AMK/GEN/AMP and AMK/CRO), β-lactam monotherapy, and fluoroquinolone-based therapy. Overall, aminoglycoside-based therapies dominated across all subgroups (62.50%), but the ESRD population demonstrated the greatest therapeutic heterogeneity.

*Enterococcus* species were identified in six patients (18.75%) with renal comorbidities, comprising two cases in glomerulonephritis (33.33%) and four in ESRD (66.67%) (Table 15). The antibiotic treatment regimens were diverse but consistently centred around combination therapies. In the GN group, management primarily included aminoglycoside–cephalosporin regimens (ERT/CFZ and CFX/AMK). Among ESRD patients, therapeutic approaches were more varied, with combinations including gentamicin–ciprofloxacin (GEN/CIP), aminoglycoside–nitrofurantoin (AMK/CN), amoxicillin-based regimens (AMC/AMPe), and ertapenem–levofloxacin (ERT/LEV). Overall, aminoglycoside-based therapies predominated, but the pattern reflected the need for broader therapeutic coverage in ESRD compared to GN.

*Pseudomonas aeruginosa* was isolated in a single patient (3.12%) with renal comorbidity, specifically in the GN group. This infection was managed using a combination regimen of cephalosporin and aminoglycoside (CFX/AMK/CAZ), reflecting the multidrug-resistant nature of this pathogen. Similarly, *Klebsiella pneumoniae* was recovered from one ESRD patient (3.12%) and was treated with a dual therapy regimen comprising ampicillin and ciprofloxacin (AMP/CIP). These findings highlight that although *Pseudomonas* and *Klebsiella* were relatively infrequent among the cohort, their presence required targeted therapeutic regimens with broad-spectrum and combination antibiotic strategies (Table 16).

## 4. Discussion

This study presents a region-specific evaluation of antimicrobial resistance (AMR) trends and uropathogen distribution in urinary tract infections (UTIs) during the late phase of the COVID-19 pandemic (2021–2022) in the Al-Baha region of Saudi Arabia. Among 1126 culture-positive urine samples, *Escherichia coli* was the predominant uropathogen, followed by *Klebsiella pneumoniae* and *Enterococcus* spp., consistent with national and international surveillance data [16,17]. The higher frequency of cases among older adults (>70 years) reinforces the well-documented link between immunosenescence, comorbidities, and increased UTI susceptibility in geriatric populations [18]. The AMR profile revealed persistently high levels of multidrug resistance (MDR) among Gram-negative organisms. Resistance to ceftriaxone in *E. coli* rose to 46.60%, while *K. pneumoniae* maintained a concerning carbapenem resistance rate of 63.60%. These figures reflect broader trends reported across tertiary centers in Riyadh and Jeddah [19]. Although this study was conducted during the late pandemic phase, the residual impact of COVID-19, particularly the empirical use of broad-spectrum antibiotics, disrupted stewardship and overwhelmed healthcare systems, may have contributed to this resistance escalation [20,21]. While carbapenems (meropenem and imipenem) and aminoglycosides (amikacin and gentamicin) maintained efficacy, resistance to fluoroquinolones, cephalosporins, and trimethoprim-sulfamethoxazole. This raises concerns about their continued use in empirical therapy, especially in community-acquired infections. These findings are consistent with international guidelines that recommend avoiding empirical use of agents with resistance rates exceeding 20.00% [16]. In contrast, Gram-positive organisms demonstrated relatively stable resistance patterns. *S. aureus* (including MRSA) and *Enterococcus* spp. remained susceptible to vancomycin, linezolid, and nitrofurantoin. These findings are supported by multicentre surveillance in Saudi Arabia and neighbouring Gulf states [18,22]. Interestingly, a slight improvement in ampicillin susceptibility among *E. coli* and aminoglycoside sensitivity in *K. pneumoniae* may indicate early benefits of reestablished antimicrobial stewardship efforts during the recovery phase of the pandemic [23].

A focused analysis of the 32 patients (2.84%) with renal comorbidities, specifically those with end-stage renal disease (ESRD), glomerulonephritis (GN), or kidney transplantation (KT), highlighted distinct microbiological and clinical patterns. While *E. coli* remained the most frequent isolate (43.80%), a disproportionately higher incidence of *S. aureus* (25.00%) and *Enterococcus faecium* (18.80%) was observed. These shifts reflect the known risk in immunosuppressed patients, who are more prone to colonization with Gram-positive organisms due to frequent catheterization, indwelling devices, and broad-spectrum antibiotic exposure [24,25]. Clinically, ESRD patients often presented with systemic symptoms, whereas kidney transplant recipients were more likely to exhibit lower urinary tract symptoms. Although these trends were not statistically significant, they are clinically meaningful and point to differing pathophysiologic mechanisms and host immune responses. Resistance rates remained high in this subgroup, even among patients receiving dual or triple antibiotic regimens, highlighting the complexity of managing UTIs in immunocompromised and catheter-dependent populations [26]. Ultimately, this study underscores the urgent need for region-specific AMR surveillance. While national networks such as SASNet provide valuable aggregated data, peripheral regions like Al-Baha are often underrepresented. By delivering updated regional insights, this study contributes to more rational empiric prescribing, especially in elderly and renal-compromised populations. Based on current susceptibility data, carbapenems and aminoglycosides remain the most reliable options for empiric therapy. Cephalosporins, fluoroquinolones, and trimethoprim-sulfamethoxazole should be reserved for culture-guided treatment.

## 5. Study Limitations

While this single-center study may limit generalizability, it addresses a critical gap in AMR surveillance for the Al Bahah region. Detailed clinical data (e.g., catheterization, diabetes, care home status) were not consistently available and thus excluded to avoid bias. However, a clearly defined renal subgroup was analyzed in depth. Phenotypic resistance profiling was used to reflect real-world diagnostics, and while conducted during the late COVID-19 phase, our findings remain relevant given ongoing changes in antibiotic use. Future research will expand clinical data collection for more stratified analysis.

## 6. Conclusions

This study provides an updated epidemiological and antimicrobial resistance (AMR) profile of uropathogens isolated at a tertiary-care hospital in the Al-Baha region of Saudi Arabia during the late phase of the COVID-19 pandemic (2021–2022). Among 1126 culture-positive UTI cases, *Escherichia coli* and *Klebsiella pneumoniae* remained the predominant pathogens. However, alarming resistance rates were observed against commonly prescribed antibiotics, including ampicillin, cotrimoxazole, fluoroquinolones, and third-generation cephalosporins. Although carbapenem and aminoglycoside resistance was comparatively lower, their continued rise is an emerging concern that warrants attention. A focused subgroup of 32 patients with renal comorbidities, including end-stage renal disease (ESRD), glomerulonephritis, and kidney transplantation, revealed a different pathogen profile, with increased prevalence of *Staphylococcus aureus* and *Enterococcus faecium*. These patients presented with more complex symptomatology and frequently required broad-spectrum or combination antimicrobial therapies. Their vulnerability underscores the clinical importance of stratified management based on comorbidity status and individualized risk assessment. Compared to pre-pandemic patterns, the findings indicate a subtle but critical escalation in AMR, likely influenced by altered healthcare and antibiotic prescribing practices during the COVID-19 pandemic. These results highlight the urgent need for strengthened antimicrobial stewardship, enhanced infection control protocols, and region-specific surveillance, particularly in underserved areas like Al-Baha. Empirical therapy for complicated UTIs in the Al-Baha region should prioritize carbapenems and aminoglycosides, especially in high-risk populations. Routine use of cephalosporins, fluoroquinolones, and cotrimoxazole should be avoided unless guided by culture data. Regionally tailored surveillance and patient care strategies are essential to combat the growing burden of multidrug-resistant UTIs in Saudi Arabia.

## Figures and Tables

**Table 1:** Correlation between gender and other demographic parameters.

Parameters		Male	Female	χ^2^ (df)	*p*-Value
		N (%)	N (%)		
Age	18–30	22 (16.79)	109 (83.21)	63.931 (5)	<0.001
	31–40	23 (15.86)	122 (84.14)		
	41–50	33 (25.00)	99 (75.00)		
	51–60	24 (25.00)	72 (75.00)		
	61–70	46 (33.82)	90 (66.18)		
	>70	209 (30.07)	486 (69.93)		
Nationality	Saudi	333 (31.50)	724 (68.50)	0.321 (1)	0.571
	Non-Saudi	24 (34.78)	45 (65.22)		
Type of patient	In-patient	181 (37.01)	308 (62.99)	11.252 (1)	<0.001
	Out-patient	176 (27.63)	461 (72.37)		
Type of cases	Recurrence	0 (0.00)	1 (100.00)	0.465 (1)	0.495
	New	357 (31.70)	768 (68.27)		

**Table 10:** Age distribution of culture-positive urinary tract infection (UTI) cases among patients with renal comorbidities.

Age Groups	Patient Diagnosis (Renal Comorbidities)			Total	χ^2^ (df)	*p*-Value
	KT	GN	ESRD			
18–30	4 (50.0)	1 (12.50)	3 (37.50)	8 (100.00)	16.90 (10)	0.076
31–40	0 (0.00)	0 (0.00)	2 (100.00)	2 (100.00)		
41–50	1 (11.11)	2 (22.22)	6 (66.67)	9 (100.00)		
51–60	0 (0.00)	0 (0.00)	2 (100.00)	2 (100.00)		
61–70	0 (0.00)	0 (0.00)	4 (100.00)	4 (100.00)		
>70	2 (28.57)	4 (57.14)	1 (14.29)	7 (100.00)		
Total	7 (21.87)	7 (21.87)	18 (56.26)	32 (100.00)		

KT = Kidney transplant, GN = Glomerulonephritis, and ESRD = End-stage kidney disease.

**Table 11:** Distribution of uropathogens isolated from culture-positive urinary tract infections (UTIs) among patients with renal comorbidities.

Uropathogens		Patient Diagnosis (Renal Comorbidities)			Total
		KT	GN	ESRD	
Gram-negative bacteria	*Escherichia coli*	5 (35.71)	3 (21.43)	6 (42.86)	14 (100.00)
	*Klebsiella pneumoniae*	0 (0.00)	0 (0.00)	1 (100.00)	1 (100.00)
	*Pseudomonas aeruginosa*	0 (0.00)	1 (100.00)	0 (0.00)	1 (100.00)
Gram-negative Sub-total		5 (31.25)	4 (25.00)	7 (43.75)	16 (100.00)
Gram-positive bacteria	*Streptococcus aureus*	1 (12.50)	1 (12.50)	6 (75.00)	8 (100.00)
	*Enterococcus faecium*	0 (0.00)	2 (33.33)	4 (66.67)	6 (100.00)
Gram-positive Sub-total		1 (14.28)	3 (21.43)	10 (64.29)	14 (100.00)
*Candida* Spp.		1 (50.00)	0 (0.00)	1 (50.00)	2 (100.00)
Total		7 (21.87)	7 (21.87)	18 (56.26)	32 (100.00)

KT = Kidney transplant, GN = Glomerulonephritis, and ESRD = End-stage kidney disease.

**Table 12:** Clinical symptoms of culture-positive urinary tract infections (UTIs) among patients with renal comorbidities.

Clinical Symptoms	Patient Diagnosis (Renal Comorbidities)			Total
	KT	GN	ESRD	
Fever	1 (10.00)	4 (40.00)	5 (50.00)	10 (100.00)
Urgency	1 (33.33)	1 (33.33)	1 (3.33)	3 (100.00)
Frequency	1 (25.00)	0 (0.00)	3 (75.00)	4 (100.00)
Dysuria	4 (26.67)	2 (13.33)	9 (60.00)	15 (100.00)
Total	7 (21.87)	7 (21.87)	18 (56.26)	32 (100.00)

KT = Kidney transplant, GN = Glomerulonephritis, and ESRD = End-stage kidney disease.

**Table 13:** Antibiotic combinations used in the treatment of culture-positive *E. coli* UTIs among patients with renal comorbidities.

Antibiotics Used for Treatment	Patient Diagnosis (Renal Comorbidities)			Total
	KT	GN	ESRD	
AMC/AMK/CTR	0 (0.00%)	0 (0.00%)	1 (100.00)	1 (100.00)
NI/CIP	0 (0.00%)	0 (0.00%)	1 (100.00)	1 (100.00)
AMK/CFZ	0 (0.00%)	0 (0.00%)	1 (100.00)	1 (100.00)
AMK/GEN/FAM	0 (0.00%)	0 (0.00%)	1 (100.00)	1 (100.00)
AMK/GEN	0 (0.00%)	0 (0.00%)	1 (100.00)	1 (100.00)
AMK/CRO/MERO	1 (33.33)	1 (33.33)	1 (33.33)	3 (100.00)
CRO/AMK/CAZ	1 (100.00)	0 (0.00%)	0 (0.00%)	1 (100.00)
AMK/CTR/CAT	1 (100.00)	0 (0.00%)	0 (0.00%)	1 (100.00)
AMK/CTR	1 (50.00)	1 (50.00)	0 (0.00%)	2 (100.00)
AMP	1 (50.00)	1 (50.00)	0 (0.00%)	2 (100.00)
AMK	0 (0.00%)	0 (0.00%)	0 (0.00)	0 (0.00)
Total	5 (35.71)	3 (21.43)	6 (42.86)	14 (100.00)

KT = Kidney transplant, GN = Glomerulonephritis, and ESRD = End-stage kidney disease.

**Table 14:** Antibiotic combinations used in the treatment of culture-positive *S. aureus* UTIs among patients with renal comorbidities.

Antibiotics Used for Treatment	Patient Diagnosis (Renal Comorbidities)			Total
	KT	GN	ESRD	
AMK/ERT	0 (0.00)	1 (50.00)	1 (50.00)	2 (100.00)
AMK/GEN/AMP	0 (0.00)	0 (0.00)	1 (100.00)	1 (100.00)
GEN/LEV	0 (0.00)	0 (0.00)	1 (100.00)	1 (100.00)
AMK/AMC	0 (0.00)	0 (0.00)	1 (100.00)	1 (100.00)
AMK/CFR/CAZ	1 (100.00)	0 (0.00)	0 (0.00)	1 (100.00)
AMK/GEN/CFR.CAZ	0 (0.00)	0 (0.00)	1 (100.00)	1 (100.00)
AMK/CRO	0 (0.00)	0 (0.00)	1 (100.00)	1 (100.00)
AMP	0 (0.00)	0 (0.00)	0 (0.00)	0 (0.00)
Total	1 (12.50)	1 (12.50)	6 (75.00)	8 (100.00)

KT = Kidney transplant, GN = Glomerulonephritis, and ESRD = End-stage kidney disease.

**Table 15:** Antibiotic combinations used in the treatment of culture-positive *Enterococcus faecalis* UTIs among patients with renal comorbidities.

Antibiotics Used for Treatment	Patient Diagnosis (Renal Comorbidities)			Total
	KT	GN	ESRD	
ERT/CFZ	0 (0.00)	1 (100.00)	0 (0.00)	1 (100.00)
GEN/CIP	0 (0.00)	0 (0.00)	1 (100.00)	1 (100.00)
AMK/CN	0 (0.00)	0 (0.00)	1 (100.00)	1 (100.00)
CFX/AMK	0 (0.00)	1 (100.00)	0 (0.00)	1 (100.00)
AMC/AMPe	0 (0.00)	0 (0.00)	1 (100.00)	1 (100.00)
ERT/LEV	0 (0.00)	0 (0.00)	1 (100.00)	1 (100.00)
Total	0 (0.00)	2 (33.33)	4 (66.67)	6 (100.00)

KT = Kidney transplant, GN = Glomerulonephritis, and ESRD = End-stage kidney disease.

**Table 16:** Antibiotic combinations used in the treatment of culture-positive *Pseudomonas aeruginosa* and *Klebsiella pneumoniae* UTIs among patients with renal comorbidities.

Bacteria Isolated	Antibiotics Used for Treatment	Patient Diagnosis (Renal Comorbidities)			Total
		KT	GN	ESRD	
*P. aeruginosa*	CFX/AMK/CAZ	0 (0.00)	1 (100.00)	0 (0.00)	1 (100.00)
*K. pneumoniae*	AMP/CIP	0 (0.00)	0 (0.00)	1 (100.00)	1 (100.00)

KT = Kidney transplant, GN = Glomerulonephritis, and ESRD = End-stage kidney disease.

## Data Availability

All data generated or analyzed during this study are included in the manuscript or its Supplementary Materials.

## Supplementary Materials

The following supporting information can be downloaded at: https://www.mdpi.com/article/10.3390/pathogens14121297/s1, Figure S1: Percentage distribution of study participants regarding their (a) gender, nationality, patient type, and types of UTI cases; (b) age of patients (N = 1126); Table S1: Sociodemographic characteristics of the study participants as per the departments attended (N = 1126).
