# Supplementary material for: Antimicrobial Resistance in Urinary Tract Infections Among Patients with and Without Renal Comorbidities: A Retrospective Study from Al-Baha, Saudi Arabia
Source: Pathogens. 2025 Dec 17;14(12):1297. doi: 10.3390/pathogens14121297 (PMC12735405; doi:10.3390/pathogens14121297)
Supplement: Supplementary file 1 [file pathogens-14-01297-s001.zip › pathogens-3993617-supplementary.pdf]

Supplementary data

Antimicrobial Resistance in Urinary Tract Infections among Patients with and without Renal Comorbidities: A Retrospective Study from Al-Baha, Saudi Arabia

Shazia Shaheen Mir*, Eman Ali, Samiyah Ahmad Abdullah Alghamdi, Nora Mohamed Alghamdi, Raed A Alharbi, Abdulmajeed A A Sindi, Ali A Zaeri

Laboratory Medicine Department, Faculty of Applied Medical Sciences, Al-Baha University, 65779, Saudi Arabia

Corresponding author*

E-mail*: smir@bu.edu.sa; Phone*: +966-557939875

Figure. S1: Percentage distribution of study participants regarding their (a) gender, nationality, patient type, and types of UTI cases, (b) age of patients (N = 1126)

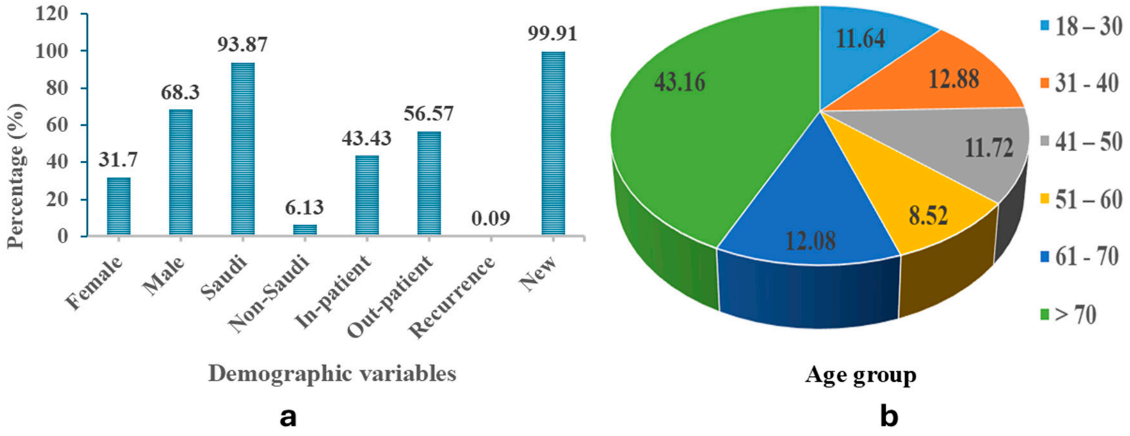

Table S1: Sociodemographic characteristics of the study participants as per the departments attended (N = 1126)

Departments	Frequency (N)	Percentage (%)
2A - SPECIAL	2	0.18
2A - SPECIAL CARE UNIT -	4	0.36
2A - SPECIAL CARE UNIT - ADULT	159	14.12
3A2 - ICU - (INTENSIVE CARE UNIT)	49	4.35
4A - PEDIA - SURGERY / MEDICAL (ALL	1	0.09
4A - PEDIA - SURGERY / MEDICAL (ALL AGES)	45	3.99
4B - PEDIA /	1	0.09
4B - PEDIA / MEDICAL (ALL	1	0.09
4B - PEDIA / MEDICAL (ALL AGES)	22	1.95
5A - MALE MEDICAL (PEDIA - ADULT)	39	3.46
5B - FEMALE MEDICAL WARD	1	0.09
5B - FEMALE MEDICAL WARD (PEDIA -	2	0.18
5B - FEMALE MEDICAL WARD (PEDIA - ADULT)	62	5.51
6A - SURGERY / ORTHO / URO	2	0.18
6A - SURGERY / ORTHO / URO (PEDIA -	1	0.09
6A - SURGERY / ORTHO / URO (PEDIA - ADULT)	21	1.86
6B - MALE SURGICAL	2	0.18
6B - MALE SURGICAL (PEDIA - ADULT)	17	1.51
7A - FEMALE SURGERY (ADULT)	27	2.4
7B - GYNE (ADULT)	28	2.48
BLOOD	1	0.09
BLOOD EXTRACTION	10	0.89
CARDIO CENTER(CLINIC)	1	0.09
CORONARY CARE UNIT (CCU)	11	0.97
DERMATOLOGY (CL INIC)	1	0.09
E.N.T(PRE- OPERATION)	1	0.09
EHC/PRE- MARRIAGE (CLINIC)	8	0.71
EMERGENCY	6	0.53
EMERGENCY CLINIC	338	30.02
ENDOSCOPY PROCEDURE	1	0.09
ER WARD	19	1.68
GEN.SURGERY- 1(CLINIC)	2	0.18
GIM (CLINIC)IMC1	8	0.71
HEMODIALYSIS UNIT	10	0.89
HOME CARE	4	0.36
INFECTIOUS DISEASE (CLINIC)-IMC1	2	0.18
LABORATORY - DEPARTMENT	17	1.51
LABOUR AND DELIVERY (ADULT)	18	1.60

MEDICATION CLINIC	6	0.53
NEPHROLOGY (CLINIC)IMC2	17	1.51
NEUROLOGY (CLINIC)IMC1	1	0.09
NEUROLOGY (SCRE ENING)	1	0.09
OB-GYNE	2	0.18
OB-GYNE (CLINIC)	31	2.75
OB-GYNE (SCREENING)	5	0.44
ONCOLOGY (CLINIC)IMC2	1	0.09
OPTHA (SCREENIN G)	1	0.09
ORTHO- 2(CLINIC)	2	0.18
PEDIA GENERAL (CLINIC)	1	0.09
PHOTOTHERAPY EVALUATION (CLINIC)	2	0.18
RADIOLOGY - DEPARTMENT	1	0.09
RHEUMATOLOGY (CLINIC)IMC2	4	0.36
UROLOGY	1	0.09
UROLOGY (ADMISSION)	13	1.15
UROLOGY (CLINIC)	50	4.44
UROLOGY (SCREENING)	31	2.75
UROLOGY ESWL (CLINIC)	9	0.8
UROLOGY SEXUAL HEALTH (CLINIC)	1	0.09
VIRTUAL DERMATOLOGY	1	0.09
VIRTUAL GIM	1	0.09
